# Supplementary material for: Experimental study on mechanical response and crack evolution law of coal and sandstone under different stress environments
Source: PLoS One. 2024 Nov 25;19(11):e0313230. doi: 10.1371/journal.pone.0313230 (PMC11588249; doi:10.1371/journal.pone.0313230)
Supplement: Attachment — Submitted filename: Response to Reviewers.docx [file pone.0313230.s001.docx]

A list of responses to reviewer comments

Manuscript ID: 24-40527

Title: Experimental study on mechanical response and crack evolution law of coal and sandstone under different stress environments

Journal: PLOS ONE

Dear editors

Thank you for kindly processing our manuscript with great patience. We have found that two reviewers raised some questions about our work. We would argue that these questions could give more benefits to improve our manuscript. The other questions are about the language, which we edited by language editing services.

In this revision stage, we have rechecked our manuscript accordingly and revised a few parts of the paper. The revision part is highlighted by *yellow background* in the revised manuscript. Our responses to the reviewers are listed in blue after each reviewer’s comments in the following pages. Please let us know if we can do anything to help facilitate the review processes. We appreciate for Editors/Reviewers’ warm work earnestly, and hope that the correction will meet with approval.

Once again, thank you very much for your comments and suggestions.

Best regards,

Qingzhao Xu

School of Mining Engineering

Anhui University of Science and Technology, Huainan, China

**Replies to reviewer #1:**

This paper studies the stability of the roadway under different stress environments and lithological conditions. The mechanical characteristics of coal rock with different intermediate principal stresses are studied by theoretical analysis and actual triaxial experiments. The influence of the change of intermediate principal stress on different lithologic materials is analyzed. The influence characteristics of the change of intermediate principal stress are obtained, and the support mode of the semi-coal rock roadway is proposed to improve the stability of the roadway. After review and study, it has a certain degree of innovation, but the article still has the following problems:

(1) The summary section suggests further condensation.

- Thank you for your valuable advice. In response to your suggestion to refine the abstract, we have summarized the description and marked it with a yellow background in the revised version. The specific changes are as follows:

In order to study the mechanical response and crack evolution law of different lithologic rock bodies under different stress environments in deep stress mines, based on the deviator stress theory, the actual triaxial disturbance unloading rock test system was used to simulate the stress occurrence environment of the original rock. The mechanical characteristics of different *σ*_2_ coal rock masses were studied, and the crack evolution law of coal and sandstone under different stress environments was analyzed. The results show that the increase of *σ*_2_ inhibits the deformation in the *σ*_2_ direction of coal and sandstone, promotes the expansion and deformation in the *σ*_3_ direction, and enhances its peak strength and elastic modulus. The development characteristics of internal cracks in rock mass are directly related to the stress environment, and the increase of *σ*_2_ promotes the increase of the proportion of coal *RA* value, weakens the proportion of sandstone *RA* value, aggravates the development of coal internal shear cracks, and inhibits the development of internal shear cracks in sandstone. The larger *σ*_2_, the greater the initial AE ringing count of coal and sandstone, and the greater the AE cumulative energy when the rock mass is finally damaged. At the same time, due to the self-organizing behavior in the process of crystal failure in sandstone, the cumulative energy curve of sandstone fluctuates in a step-like manner. The ringing count and cumulative energy increase suddenly, which can predict the imminent instability and failure of the rock, and the research results can provide an experimental basis for the sudden instability of deep high-stress roadways.

(2) What is the basis for the design of the stress field in the test? How are the numbers considered? Please explain.

- Thank you for your advice. In response to your question about how the stress field is set in the article, we have made additional changes in the revised manuscript and marked it with a yellow background. For specific changes, please refer to the section of the experimental protocol. The specific changes are as follows:

According to the schematic diagram of the stress distribution of the surrounding rock of the roadway (Fig.4), it can be seen that due to the change of the principal stress in the process of excavation of the roadway, as the rock mass is far away from the free side, the horizontal stress shows a transition from the direction of stress increase - stress reduction - original stress, at this time, the maximum principal stress is the vertical principal stress (considering the buried depth is 680m, the vertical principal stress *σ*_1_=17MPa), the position of the unit body is selected in the roadway gang and near the free side, so the minimum principal stress *σ*_3_=2MPa is set, and at the same time, The rock mass at the roadway excavation face has no supporting effect, which forces the rock mass in front of it to produce slow deformation behavior, resulting in changes in the horizontal stress at different positions of the rock mass in front of the roadway (intermediate principal stress *σ*_2_). Therefore, to study the influence of different intermediate principal stresses on the failure and deterioration mechanism of the surrounding rock mass, the intermediate principal stresses *σ*_2_=3MPa, 6MPa, 10MPa, and 14MPa are set.

**Fig.4 Schematic diagram of stress distribution in surrounding rock of roadway**

(3) The analysis of Figure 6 is not thorough enough, and the main reasons for the different changes between the two need to be further analyzed.

- Thank you for your advice. In response to your question, we have performed a redescription analysis and marked the revised manuscript with a yellow background. The specific changes are as follows:

In order to analyze the influence of *σ*_2_ on the lateral peak strain of coal and sandstone, the lateral peak strain corresponding to different *σ*_2_ was plotted (Fig.7). It can be seen from Fig.7 that with the increase of *σ*_2_, the strain of coal and sandstone in the *ε*_2_ direction decreases. The peak strain of coal and sandstone in the *ε*_3_ direction increases, and the change of the peak strain of coal *ε*_3_ is higher than that of sandstone due to the properties of the rock mass itself. The peak strain of coal at the peak of *ε*_2_ is higher than that of sandstone before *σ*_2_ =6MPa, and the peak strain of coal is lower than that of sandstone after 6MPa. The reason for the analysis is that the coal body is not dense; there are many internal primary pores and fractures, and compared with the sandstone, it presents a complex structure, and the coal is prone to fractures and expansion under the action of the same lateral horizontal principal stress. With the development of fractures, the ability to connect the internal structures is further weakened, resulting in the aggravation of deformation. Compared with sandstone, the rate of fracture propagation under stress is slower, and the deformation is more limited, so the growth of lateral strain is less than that of coal.

(4) It is proposed that sections 4.1 and 4.3 be combined to reorganize the analysis.

- Thank you for your advice. In response to your question, we make the following explanations: in the analysis of the experimental results, we first analyze the stress-strain curves of coal and sandstone to obtain the characteristics of the stress-strain curves of the two, secondly, according to their peak strength and elastic modulus, obtain the response relationship between the peak strength and elastic modulus of the two and the intermediate principal stress, and finally comprehensively sort out the fundamental mechanical characteristics of the stress-strain curve, peak strength and elastic modulus—a more comprehensive analysis of the main factors that led to the macro disruption of both. Therefore, the logical order of the articles will be more precise by analyzing them separately.

(5) It is mentioned in many places in the article that with the increase of the second principal stress, it is too verbose, and it is recommended to delete the irrelevant text and re-summarize the conclusion.

- Thank you for your advice. In response to your question, we have made changes in the revised manuscript and marked the changes with a yellow background. The specific changes are as follows:
- In this paper, the theory of deviator stress is introduced to analyze the influence of deviator stress on the plastic zone of the roadway. A triaxial testing machine studies the mechanical properties and acoustic emission evolution law of coal and sandstone with different intermediate principal stresses. The standard and different characteristics of different lithologies under the same stress are analyzed, and the following conclusions are obtained:
- (1) The increase of *σ*_2_ inhibits the development of strain in the *σ*_2_ direction of coal and sandstone, promotes the increase of strain in the *σ*_3_ direction, and gradually increases the induced lateral strain difference. Its peak strength and elastic modulus are positively correlated with *σ*_2_. However, the amplification effect is different; the peak strength and elastic modulus of sandstone increase by 40% and 76% from *σ*_2_=3MPa to *σ*_2_=14MPa, respectively, while the peak strength and elastic modulus of coal increase by 37% and 44%, respectively.
- (2) The ringing count and cumulative energy generated by the acoustic emission at the peak of coal and sandstone are positively correlated with *σ*_2_. Due to the different lithologies, the coal acoustic emission ringing count occurs frequently, the value is significant, and the cumulative energy of the acoustic emission is low. In contrast, the sandstone acoustic emission ringing count is small; the value is small. The cumulative energy of the acoustic emission is significant, and the cumulative energy of the coal acoustic emission gradually increases without the ladder phenomenon. The cumulative energy of sandstone acoustic emission appears to have many apparent steps.
- (3) With the increase of *σ*_2_, there are apparent differences in the failure characteristics of different lithologies under the same *σ*_2_. The proportion of *RA* value of sandstone decreased by 37.38% from 75.62% to 38.24%, and the failure characteristics changed from the primary shear failure to the tensile and shear composite failure mode. However, with the increase of *σ*_2_, the development of shear cracks was intensified, and the proportion of *RA* value increased by 3.11% from 67.99% to 71.10%. The characteristics of shear failure became more and more evident during failure.
- (4) Under the same stress environment, there are apparent differences in the mechanical properties and crack development of different lithology rocks. When the roadway is a semi-coal rock roadway, the roadway often fails in the weak rock mass. At this time, the weak rock mass is strengthened. Support and weaken the risk of uncoordinated deformation and failure due to different lithological rock masses to enhance the overall stability of the surrounding rock of the roadway.

(6) Starting from the stress field of the surrounding rock, the stress state of the rock mass under the actual triaxial stress state is introduced, and the clarification sequence is reasonable and smooth, but the literature in the past five years accounts for a small proportion of the references, so it is recommended to update further.

- Thank you for your advice. In response to your suggestion to add references from the last five years, we have made changes in the revised manuscript and marked the changes with a yellow background.

**Replies to reviewer #2:**

Authors have conducted experimental study on mechanical response and crack evolution law of coal and sandstone under different stress environments. Before the manuscript can be recommended to be accepted, authors have to make some major revisions as follows:

(1) Language and grammar errors: L39 “presents the complex stress characteristics”, this sentence lacks a subject. L77 “…for acoustic emission Chen…”

- Thank you for your advice. In response to your question, we have asked native English speakers to re-edit the full text of the sentence and mark it with a yellow background in the revised manuscript.

(2) In section Experimental scheme: a) More information about research objects should be provided such as sampling location of rock, basic properties and so on. b) Why the default value of the maximum principal stress *σ*_1_ is 17MPa and the minimum principal stress *σ*_3_ is 2MPa? How are the four different values of intermediate principal stress selected? It is suggested supply experiment design basis in this section.

- Thank you for your advice. In response to your question, we have supplemented the protocol section of the article and marked it with a yellow background. The specific changes are as follows:
- The coal and sandstone required for this test come from the Guqiao Coal Mine of Anhui Huainan Mining Group Co., Ltd. The large coal and sandstone body taken on site are processed into a cube shape of 100mm×100mm×100mm (unevenness ≤0.02mm, size error ≤0.2mm) by laboratory means to ensure that the accuracy of the sample meets the requirements, the specific coal and sandstone samples are shown in Fig.3, and the uniaxial compressive strength of the test coal is 21.665MPa, The Poisson's ratio is 0.155, the uniaxial compressive strength of sandstone is 42.608MPa, and the Poisson's ratio is 0.059. The triaxial servo-controlled testing machine preloads the specimen in three directions simultaneously. The preset pressure values in the three directions are reached simultaneously. Then, σ3 and σ2 remain unchanged, and σ1 increases at 30kN/min until the sample is damaged and unstable. According to the schematic diagram of the stress distribution of the surrounding rock of the roadway (Fig.4), it can be seen that due to the change of the principal stress in the process of excavation of the roadway, as the rock mass is far away from the free side, the horizontal stress shows a transition from the direction of stress increase - stress reduction - original stress, at this time, the maximum principal stress is the vertical principal stress (considering the buried depth is 680m, the vertical principal stress *σ*_1_=17MPa), the position of the unit body is selected in the roadway gang and near the free side, so the minimum principal stress *σ*_3_=2MPa is set, and at the same time, The rock mass at the roadway excavation face has no supporting effect, which forces the rock mass in front of it to produce slow deformation behavior, resulting in changes in the horizontal stress at different positions of the rock mass in front of the roadway (intermediate principal stress *σ*_2_). Therefore, to study the influence of different intermediate principal stresses on the failure and deterioration mechanism of the surrounding rock mass, the intermediate principal stresses *σ*_2_=3MPa, 6MPa, 10MPa, and 14MPa are set.

**Fig.4 Schematic diagram of stress distribution in surrounding rock of roadway**

(3) In L211, it would be good to supply references to prove this research results are in good agreement with the existing research.

- Thank you for your advice. In response to your question, we have reviewed the relevant references and introduced them into the text, marked with a yellow background.

(4) Conclusions: it is suggested to add one more paragraph introducing the methodology briefly employed in this work, before drawing specific conclusions.

- Thank you for your advice. In response to your question, we have added it to the article and marked it with a yellow background. The specific changes are as follows:
- In this paper, the theory of deviator stress is introduced to analyze the influence of deviator stress on the plastic zone of the roadway. A triaxial testing machine studies the mechanical properties and acoustic emission evolution law of coal and sandstone with different intermediate principal stresses. The standard and different characteristics of different lithologies under the same stress are analyzed, and the following conclusions are obtained:
